# Positron Emission Tomography in the Diagnosis and Management of Coronary Artery Disease

**DOI:** 10.3390/medicina54030047

**Published:** 2018-07-11

**Authors:** Eglė Kazakauskaitė, Diana Žaliaduonytė-Pekšienė, Eglė Rumbinaitė, Justas Keršulis, Ilona Kulakienė, Renaldas Jurkevičius

**Affiliations:** 1 Department of Cardiology, Medical Academy, Lithuanian University of Health Sciences, Kaunas LT-50161, Lithuania; Diana.Peksiene@kaunoklinikos.lt (D.Ž.-P.); egle.rumbinaite@gmail.com (E.R.); justas.kersulis@gmail.com (J.K.); Renaldas.Jurkevicius@kaunoklinikos.lt (R.J.); 2 Department of Radiology, Medical Academy, Lithuanian University of Health Sciences, Kaunas LT-50161, Lithuania; kulakiene@dr.com

**Keywords:** positron emission tomography, coronary artery disease, myocardial viability, myocardial perfusion imaging

## Abstract

Cardiac positron emission tomography (PET) and positron emission tomography/computed tomography (PET/CT) are encouraging precise non-invasive imaging modalities that allow imaging of the cellular function of the heart, while other non-invasive cardiovascular imaging modalities are considered to be techniques for imaging the anatomy, morphology, structure, function and tissue characteristics. The role of cardiac PET has been growing rapidly and providing high diagnostic accuracy of coronary artery disease (CAD). Clinical cardiology has established PET as a criterion for the assessment of myocardial viability and is recommended for the proper management of reduced left ventricle (LV) function and ischemic cardiomyopathy. Hybrid PET/CT imaging has enabled simultaneous integration of the coronary anatomy with myocardial perfusion and metabolism and has improved characterization of dysfunctional areas in chronic CAD. Also, the availability of quantitative myocardial blood flow (MBF) evaluation with various PET perfusion tracers provides additional prognostic information and enhances the diagnostic performance of nuclear imaging.

## 1. Introduction

Despite considerable advances in prevention and management of atherosclerotic heart disease and its devastating consequences, the morbidity and mortality of coronary artery disease (CAD) [1] in developed countries remains high [2]. This review presents an overview of the place of cardiac positron emission tomography (PET) in the diagnosis and management of CAD.

Since the first PET scanners were introduced into a daily routine more than thirty years ago [3], they have been used for the non-invasive assessment of various cardiac conditions [4]. PET allows functional imaging of the heart, as opposed to anatomical imaging by other non-invasive cardiovascular imaging methods like cardiac computed tomography angiography (CCTA) and magnetic resonance imaging (MRI) [5]. In clinical cardiology, PET is established as the best available test not only for the assessment of myocardial viability but also for the evaluation of myocardial perfusion, as the standard for validation of many new techniques. [6,7,8]. Nevertheless, there are several factors limiting the integration of cardiac PET into clinical routine (difficult production of positron-emitting radioligands of short physical half-life (Table 1), generation of perfusion tracers which need an on-site cyclotron facility and the high PET imaging costs) [4].

## 2. Positron Annihilation

**Figure 1:**
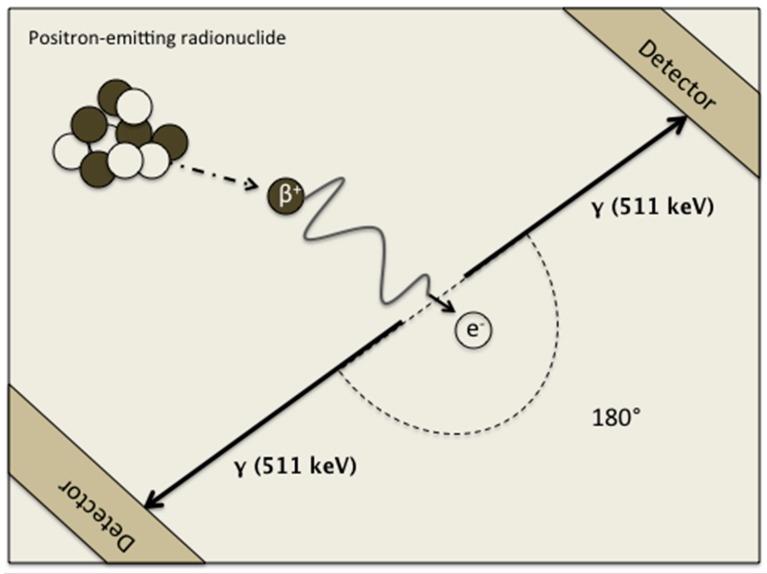
Simplified schematic overview of positron annihilation.

PET is a non-invasive method that produces images of a radionuclide-labelled tracer distribution in the body [9]. PET imaging, as the name suggests, is based on positron emission by the decaying radionuclide (Figure 1). As the elementary particle of antimatter, a positron is an equivalent of an electron. Being of the same mass but of the opposite electrical charge, it is released from a proton inside the nucleus, to collide with nearby electrons [9]. In a positron-electron annihilation reaction it forms a pair of gamma-ray photons traveling in opposite directions and having energy of 511 keV. Gamma rays can be detected in coincidence by opposing detectors. PET imaging technique provides high spatial resolution (4 mm) that makes PET superior compared to other conventional nuclear techniques [9].

## 3. Radiopharmaceuticals

The dominant applications of radiopharmaceuticals in clinical cardiology can be classified into two main categories: studies of myocardial metabolism (viability) and the assessment of regional myocardial blood flow (MBF). Table 1 summarizes the most commonly used cardiac radiotracers.

**Figure 2:**
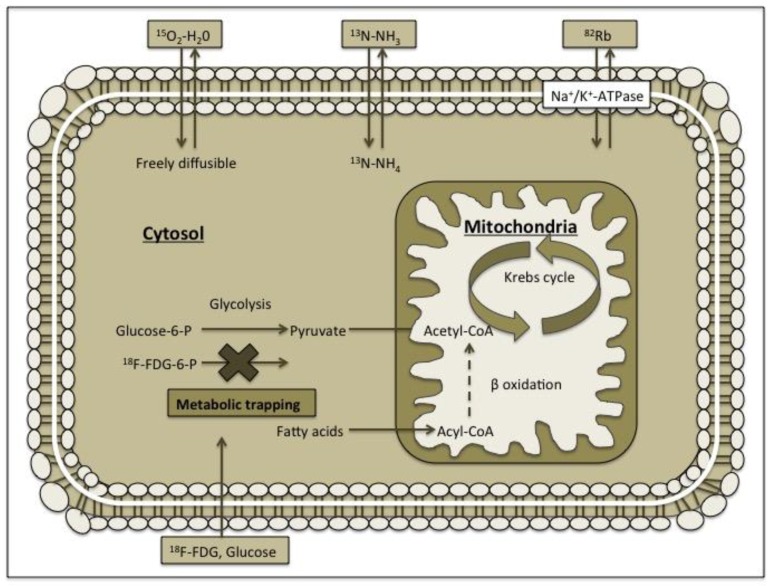
Mechanism of action of different positron-emission tomography (PET) tracers. Fludeoxyglucose (FDG) is a biological analogue of glucose but unlike glucose it is intracellularly trapped and does not undergo further metabolism. ^15^O-water is metabolically inert and freely diffusible perfusion tracer. ^82^Rb is the perfusion tracer that is taken up by myocardium as a potassium analogue through the active transport by the Na+/K+ ATPase-pump. ^13^N-NH_3_ is taken up by the myocardium by passive free diffusion across cell membranes as ammonia (NH_3_) where it equilibrates with its charged form ammonium (NH_4_).

^18^F-FDG (fludeoxyglucose F) is established as the myocardial viability tracer [10,11,12]. Fludeoxyglucose (FDG) is a glucose analogue but, unlike glucose, it is being intracellular trapped and does not undergo further metabolism (Figure 2). In chronically under-perfused but viable myocardium, there is an increased FDG uptake caused by up-regulation of anaerobic pathways, while in scars, FDG uptake is decreased or even absent [13,14]. To increase myocardial glucose uptake and to improve image quality, the standardized patient preparation protocols are used (hyperinsulinemic euglycemic clamping, oral glucose loading and administration of nicotinic acid derivatives) [15,16]. Also, because ^18^F nuclide has a rather long physical half-life (Table 1), it is possible to produce FDG centrally and distribute in smaller centres without a need of on-site cyclotron.

Assessment of myocardial perfusion is one of the major PET clinical applications. For the evaluation of MBF mainly three tracers are used: ^15^O-labelled water (^15^O-H_2_O) [17,18] ^13^N-labelled ammonia (^13^NH_3_) [19,20] and the cationic potassium analogue ^82^Rubidium (^82^Rb) [21]. In addition, there is a novel promising ^18^F-labelled PET perfusion tracer ^18^F-BMS747158-02 (Flurpiridaz F18) that has been recently validated in animals [22] and humans [23,24].

**Figure 3:**
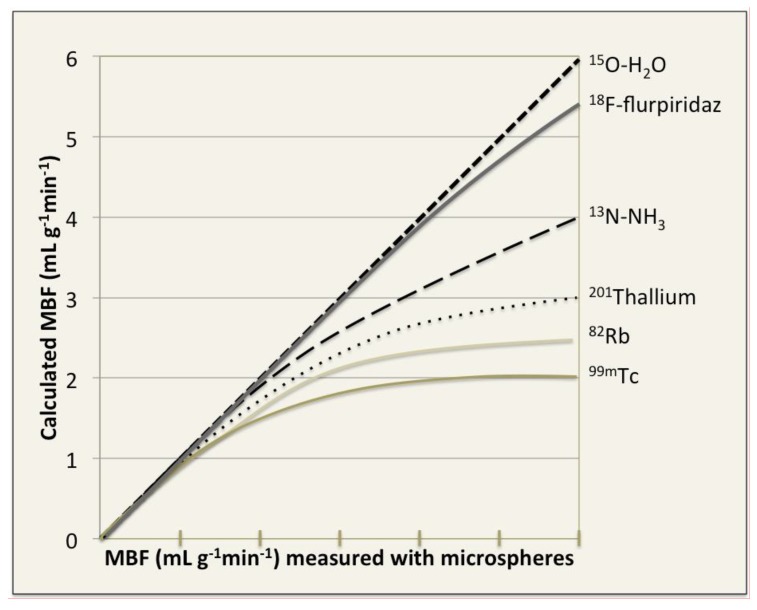
Simplified schematic representation of radiotracers extraction. ^15^O-H_2_ O is the only perfusion tracer with a linear relationship between myocardial extraction and myocardial blood flow (MBF). ^13^N-NH_3_ has linear myocardial uptake over a wide range of MBF, except the very high flow rates, while the extraction of all other tracers (PET and single photon emission computed tomography (SPECT) perfusion tracers) tends to stabilize at increasing flow values. However, novel PET perfusion tracer ^18^F-flurpiridaz is demonstrating important properties that an ideal PET myocardial perfusion imaging (MPI) radiotracer contains and firstly it is a high myocardial extraction fraction.

Radiolabelled water (^15^O-H_2_O) has been widely used for the experimental issues [25,26,27,28,29]. ^15^O-water is metabolically inert and freely diffusible perfusion tracer (Figure 2) with a very short half-life (2.06 min) [30] (Table 1). Of all perfusion tracers only radiolabelled water shows a linear relationship between myocardial extraction and MBF and allows accurate quantifying of MBF over a wide range of values (Figure 3, Table 2) [31,32,33]. However, ^15^O-H_2_O quickly reaches equilibrium between blood pool and myocardium and can thereby interfere with appropriate images of myocardial perfusion.

^82^Rb is the perfusion tracer that is taken up by myocardium as a potassium analogue via active transport by the Na+/K+ ATPase-pump (Figure 2) [34]. ^82^Rb has a short physical half-life (1.25 min) [30], however, an on-site cyclotron is not needed, since ^82^Rb can be produced by the ^82^Sr/^82^Rb generator that generally needs replacement once a month (Table 1). Nevertheless, myocardial perfusion images with ^82^Rb can be underestimated because of its long positron track resulting in a reduced spatial resolution and a nonlinear relation between myocardial extraction and MBF (Figure 3, Table 2) [35,36]. Despite the clear limitations, ^82^Rb provides useful information in the clinical setting and is used extensively for the evaluation of myocardial perfusion [37,38,39,40].

^13^N-NH_3_ is taken up by myocardium by passive free diffusion across cell membranes as ammonia (NH_3_), where it equilibrates with its charged form ammonium (NH_4_) (Figure 2) and gets trapped inside the cell by conversion through glutamine synthase to ^13^N-glutamine [34]. ^13^N-ammonia has a relatively short physical half-life (9.96 min) [23] (Table 1) and requires an on-site cyclotron facility. However, ^13^N-NH_3_ has linear myocardial uptake relationship with MBF, except the very high MBF rates (Figure 3) [41,42,43], which leads to high accuracy of perfusion images and regional MBF quantification. Furthermore, the length of its half-life is sufficient for the stress test feasibility (Table 2) [44]. The mentioned above features make ^13^N-ammonia the best choice for the myocardial perfusion imaging in PET centres with available cyclotron facility.

^18^F-BMS747158-02 (flurpiridaz) is an analogue of the insecticide pyridaben with a good uptake in the heart because of high affinity for complex I of the mitochondrial electron transport chain [45]. The available evidence of this novel perfusion tracer indicates high suitability of ^18^F-flurpiridaz for perfusion imaging. The half-life of this tracer is relatively long (109 min), therefore, the on-site cyclotron is not necessary. In pre-clinical and clinical trials, ^18^F-flurpiridaz has exhibited properties of a perfect PET perfusion radiotracer: a high myocardial extraction fraction (Figure 3, Table 2), high resolution images of defects, high and stable myocardial-to-background contrast, good myocardial uptake and slow clearance [30]. In the phase II study [24], ^18^F-flurpiridaze PET appeared safe with high image quality and therefore increased interpretative certainty and overall diagnostic performance for CAD. Nevertheless, to assess the real potential of ^18^F-flurpiridaz larger studies are necessary. In recent years, several other ^18^F labelled cardiac PET perfusion tracers have been reported (*p*-fluorobenzyl triphenyl phosphonium cation (^18^F-FBnTP), 4-fluorophenyl triphenyl phosphonium ion (^18^F-FTPP) and fluorodihydrorotenone (^18^F-FDHR) [46,47,48]; however, currently only ^18^F-flurpiridaz has reached advanced clinical evaluation with promising results [49,50,51].

## 4. Assessment of Myocardial Viability with Positron Emission Tomography (PET)

There are two ways that dysfunctional but viable myocardium can exist [52]. Myocardial stunning is known as the phenomenon in patients with CAD when myocardial contractility is reduced for several hours due to the short nonlethal episodes of ischemia but finally normal perfusion is fully restored [53]. Hibernating myocardium is known as the consequence of repetitive ischemia due to normally increased myocardial metabolic demand when a significant stenosis and severely limited coronary flow reserve (CFR) are present [14]. Contractility is recovered spontaneously and, in order to restore adequate CFR, coronary revascularization needs to be performed [54].

**Figure 4:**
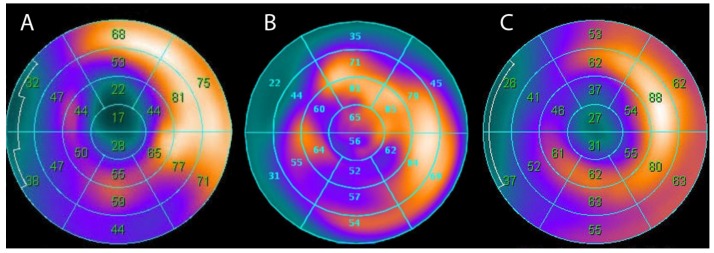
Clinical example of “flow-metabolism mismatch.” 76 years old male with a clinical history of anterior myocardial infarction was admitted to the hospital due to unstable angina. Coronary angiography revealed triple coronary artery disease. Myocardial perfusion imaging (MPI) (99mTc-MIBI SPECT at rest) (**A**) demonstrated perfusion defect in apex and apical septal, anterior and lateral segments, while myocardial viability test (FDG-PET) (**B**) in the same segments revealed viable myocardium. Surgical revascularisation was performed. Six months after coronary artery bypass grafting (CABG) patient‘s functional class was increased, he had no angina and MPI (99mTc-MIBI SPECT at rest) (**C**) revealed slightly better perfusion in the same segments.

Because of the high perioperative or peri-interventional risk for CAD patients with decreased LV function, it is extremely important to distinguish patients who can potentially benefit from the revascularization, particularly those patients with viable myocardium. Several techniques can be used to assess viable myocardium: dobutamine stress or myocardial contrast echocardiography, ^201^TI SPECT, nitrate augmented MPI, FDG PET and delayed enhancement MRI [55]. Nevertheless, as it was mentioned previously, a non-invasive PET modality has become a standard for both myocardial perfusion and myocardial viability assessment. [6,7,8]. Comparing FDG images with the perfusion images (e.g., ^13^N-NH_3_ PET, ^99m^Tc-MIBI, ^99m^Tc-Tetrofosmin or ^201^TI SPECT), it is possible to distinguish viable myocardium from a fibrotic scar (with no possibility of functional recovery). Regions with reduced perfusion and FDG uptake are considered as irreversibly injured and without viability (“flow-metabolism match”), whereas reduced perfusion regions with relatively preserved FDG uptake (“flow-metabolism mismatch”) are considered to be viable (Figure 4).

Recent studies suggested that myocardial viability is considered as an independent predictor of cardiac death in ischemic cardiomyopathy patients if they were treated medically [56].

However, in chronic ischemic heart disease (IHD), three prospective randomized trials, the PET and recovery following revascularization (PARR-2) trial [12], the heart failure revascularization (HEART) trial [57] and the surgical treatment for ischemic heart failure (STICH) trial [58] failed to show a clear survival benefit of revascularization in the setting of viable myocardium over optimal medical treatment alone. These results are still debated widely [59,60]. Methodological limitations of the trials are significant; additionally, meta-analyses of pooled data from the single centre studies are also conflicting [60,61]. However, recent heart failure guidelines of European Society of Cardiology [62] recommended consideration of non-invasive stress imaging for the assessment of both inducible ischemia and viable myocardium in patients with heart failure (HF) and CAD (considered suitable for coronary revascularization) before the decision on revascularization [58,61,63]. Bax et al. suggested that a significant improvement of left ventricle (LV) ejection fraction (EF) (>5%) after revascularization can be obtained when a critical amount of at least four dysfunctional but viable segments are present [64]. Other studies demonstrated a direct relationship between the magnitude of recovery of LV EF after revascularization and the number of dysfunctional viable segments by PET [65]. Ling et al. concluded that in the setting of significant hibernating myocardium (extent of viability exceeded 10% of the myocardium), early revascularization was related with increased survival compared to medical therapy alone [63]. A recent study, performed by Namdar et al., estimated the prognostic value of FDG-PET/CT in elderly patients with stable coronary artery disease (CAD) and reduced left ventricular ejection fraction (rLVEF) before revascularisation. This PET/CT study revealed that FDG PET/CT detected viable myocardium was associated with better clinical outcomes in elderly patients when revascularised [66].

## 5. PET Perfusion Imaging and Myocardial Blood Flow

After decades of experience with scintigraphic assessment of myocardial perfusion, myocardial perfusion imaging (MPI) has been validated for the prognosis of cardiac disease and the technique embedded in national and international guidelines. PET has been widely used for myocardial viability assessment but now it is being increasingly applied to detect flow-limiting CAD.

Clinical manifestation of CAD is symptoms caused by myocardial ischemia. Nevertheless, significant part of CAD includes asymptomatic subclinical stages, such as functional vascular alteration (endothelial and microvascular dysfunction), which PET allows to detect in patients at risk of developing CAD [4]. PET leads to diagnosis and prognostication of established or suspected CAD patients, while in patients with known ischemic myocardial dysfunction, it facilitates stratification.

PET appears to be a superior myocardial perfusion technique compared to the widely used SPECT, as its spatial resolution is higher and there are methods of attenuation correction available. The average sensitivity and specificity, comparing PET with invasive coronary angiography, was 93% and 92%, respectively [67]. PET has higher specificity due to the reduced number of artefacts related to photon attenuation, allowing a decrease of false positive findings (particularly in obese patients) [67]. The higher sensitivity of PET may be attributed to a higher spatial resolution as well as to a higher tracer uptake in myocardium at the high flow rates. Nevertheless, a close comparison of PET with SPECT is lacking [4].

Symptomatic CAD at early stages and/or asymptomatic CAD can be evaluated with PET assessing an absolute MBF in millilitres per gram per minute [68]. For coronary blood flow measurements, there are some other techniques available (e.g., Doppler catheterization); however, these invasive methods do not provide values of volumetric flow to myocardium as they measure coronary flow velocity (in cm/sec) [4]. Some studies revealed non-invasive PET as an accurate tool that applies suitable tracers and mathematical models for the quantification of regional MBF [33]. Coronary vascular function assessment by PET is particularly helpful in patients with intermediate cardiovascular risk [69,70,71]; the detection by PET of functional abnormalities of coronary vessels could be superior to detection of alterations in arterial wall structure and may identify the functional lesion before the structural changes appear.

PET assessment of MBF allows [68]:

1. Identification of subclinical CAD. SPECT myocardial perfusion imaging is a traditional evaluation of symptomatic patients with known or suspected CAD [71,72,73]. However, the early stages with the functional microvascular dysfunction remain undetected with SPECT but can be identified by PET by quantifying hyperaemic MBF or MBF in absolute terms [1,69,74,75,76].
2. Enhanced characterization of CAD burden and identification of “balanced” decrease of MBF in all vascular territories. Patients with multivessel CAD may show normal SPECT results due to an absolute decrease of radiotracer uptake in the left ventricle (LV) myocardium. Previous studies have disclosed that SPECT perfusion imaging can detect approximately only 10% of patients with severe three-vessel CAD or significant left main coronary artery (LMA) stenosis (≥50%) [77,78]. If the gated SPECT images are applied, the identification of these patients can be improved to 25% [79]. In diffuse atherosclerosis, PET can identify the hemodynamically significant culprit single lesion along with the true extent of ischemia in a multivessel territory [80,81].

The coronary flow reserve (CFR) is a ratio of near-maximal coronary vasodilatation MBF to basal MBF and is known as an indirect parameter to evaluate the function of the coronary circulation (not only the possible narrowing of the epicardial coronary arteries but also the dysfunction of coronary microcirculation when the angiographic evidence of CAD is absent). Additionally, endothelium-dependent vasoreactivity (e.g., using the cold pressor test) also can be evaluated by PET, providing an estimate of endothelial integrity, which can be reduced in patients with coronary risk factors [82], such as arterial hypertension [83], diabetes [84], hypercholesterolemia and smoking [27]. Tio et al. have shown that, in medically treated patients with ischemic cardiomyopathy and reduced LV function, an abnormal CFR (measured with ^13^N-NH_3_ PET) is a strong and independent prognostic factor of cardiac mortality (hazard ratio 4.11; CI 95%, 2.98–5.67, per standard deviation of CFR) [85]. This and other studies suggest that regardless of the presence or absence of detectable CAD, the PET evaluation of vasodilator microcirculation capacity may provide additional prognostic information.

## 6. Integration or Fusion of Different Imaging Modalities

Over the past several years, there has been a substantial development in combination of PET with computed tomography (CT) or MRI. Such fusion of different imaging modalities can supply complementary data in a single examination [86] and allows leveraging the strengths of each modality, improves the diagnosis, management and prediction of clinical outcomes of various cardiovascular diseases and, firstly, CAD.

Several studies have investigated PET/CT benefit in suspected CAD patients [87,88]. Valenta et al. have demonstrated better understanding of the hemodynamic significance of coronary stenosis in the PET/CT evaluation of 24 patients (^13^N-ammonia was used to assess different variables of myocardial blood flow: flow reserve and flow gradients, which were related with computed tomography coronary angiography (CTCA)) [87]. Dey et al. designed a study with 51 patients who underwent PET/CT hybrid examination: CTCA (quantitatively analysed) was combined with myocardial flow reserve (obtained from rest-stress ^13^N-ammonia PET) [88]. The reduced myocardial flow reserve (indicating ischemia) Optimal prediction of was predicted by severity of CTCA stenosis integrated with other CTCA variables (including total plaque burden). Both studies demonstrated better PET/CT hybrid prediction of hemodynamically significant coronary stenosis compared to PET or CTCA alone.

PET/MRI was originally developed for neuroimaging [89,90] and, despite the technical difficulties, in the recent years has become an exciting cardiovascular diagnostic tool. The major progress came when photodiode and silicon photomultiplier detectors were adapted to work with the MRI scanner [91,92]. These technical innovations led to the development of a truly hybrid system within the same gantry, including MR and PET modalities (Biograph mMR, Siemens Healthcare, Erlangen, Germany; Signa PET/MR, GE Systems, Waukesha, Wisconsin) [93]. Cardiac MRI excellently characterizes soft tissues and would complement molecular imaging of PET in understanding myocardial viability after infarction, ventricular remodelling, infiltrative diseases and inflammatory processes in particular. Although, PET/MRI hybrid scan is complex and expensive, the single scan combines the strengths of two already powerful and individual imaging modalities, while providing additional advantages, such as reduced radiation exposure, motion and partial volume correction, superior soft tissue contrast and multiparametric multiorgan assessments [93]. Nevertheless, the technical challenges need to be solved and further studies performed in the PET/MRI area for the better concept of potential future applications. As example, the recent initial clinical validation of Motion-corrected whole-heart PET-MR for the simultaneous visualisation of coronary artery integrity and myocardial viability [94], should be mentioned. Munoz et al. revealed that motion correction increased visible length and sharpness of the coronary arteries by CMRA (coronary MR angiography) and improved delineation of the myocardium by 18F-FDG PET, resulting in good agreement with X-ray angiography and LGE (Late Gadolinium Enhancement) -MRI [94].

## 7. Conclusions

Cardiac PET and PET/CT are encouraging and precise non-invasive imaging modalities that allow functional imaging of the heart, while other non-invasive cardiovascular imaging modalities are considered to be techniques of anatomical imaging. The role of cardiac PET is growing rapidly as it allows diagnosing CAD with high accuracy. In clinical cardiology, PET is established as the best available technique for evaluation of myocardial viability and is recommended for the proper management of reduced LV function and ischemic cardiomyopathy.

Hybrid PET/CT imaging has enabled simultaneous integration of coronary anatomy and function with myocardial perfusion and metabolism in the same examination and has improved characterization of the dysfunctional areas in chronic CAD. Also, the availability of quantitative MBF evaluation with various PET perfusion tracers provides additional prognostic information and increases diagnostic performance in CAD management. However, there are no ideal imaging modalities yet that could offer an accurate picture of complex pathophysiological substrate of LV dysfunction and the potential for its reversibility. Revascularization decisions in cases of ischemic LV dysfunction continue to be individual and complicated, demanding integration of different clinical and anatomic imaging modalities. This leads to a conclusion that the future cardiac PET research lies in the further development and understanding of molecular imaging, as well as in the investigation and establishment of new perfusion tracers suitable for PET imaging in diagnostic centres without an on-site cyclotron facility.

## Figures and Tables

**Table 1:** The most common cardiac positron emission topography (PET) tracers.

Tracer	Physical Half-Life	Uptake Mechanism	Radionuclide Production	Cyclotron on/off-Site
^15^O-H_2_O	2.06 min	Free diffusion (perfusion)	Cyclotron	on-site
^82^Rb	1.25 min	Na/K-ATPase (perfusion)	^82^Sr/^82^Rb generator	off-site
^13^N-NH_3_	9.96 min	Diffusion/metabolic trapping (perfusion)	Cyclotron	on-site
^18^F-FDG	109 min	Glucose transport/hexokinase (viability)	Cyclotron	off-site

**Table 2:** Characteristics of cardiac PET perfusion tracers.

	^15^O-H_2_0	^13^N-NH_3_	^82^Rb	^18^F Flurpiridaz
Positron range (mm)	4.14	2.53	8.6	1.03
Image resolution	Intermediate	Intermediate-high	Lowest	Highest
Myocardial extraction fraction	100%	80%	65%	94%
Pharmacological stress imaging protocol	Feasible	Feasible	Feasible	Feasible
Treadmill exercise Imaging protocol	Not feasible	Feasible but not practical	Not feasible	Feasible

## References

[1] Schindler T.H., Facta A.D., Prior J.O., Cadenas J., Zhang X.L., Li Y., Sayre J., Goldin J., Schelbert H.R. (2009). Structural alterations of the coronary arterial wall are associated with myocardial flow heterogeneity in type 2 diabetes mellitus. Eur. J. Nucl. Med. Mol. Imaging.

[2] Vedanthan R., Fuster V. (2009). Disease Prevention: The moving target of global cardiovascular health. Nat. Rev. Cardiol..

[3] Ter-Pogossian M.M., Phelps M.E., Hoffman E.J., Mullani N.A. (1975). A positron-emission transaxial tomograph for nuclear imaging (PETT). Radiology.

[4] Gaemperli O., Kaufmann P.A. (2011). PET and PET/CT in cardiovascular disease. Ann. N. Y. Acad. Sci..

[5] Townsend D.W. (2008). Positron emission tomography/computed tomography. Semin. Nucl. Med..

[6] Klein C., Nekolla S.G., Bengel F.M., Momose M., Sammer A., Haas F., Schnackenburg B., Delius W., Mudra H., Wolfram D. (2002). Assessment of myocardial viability with contrast-enhanced magnetic resonance imaging: Comparison with positron emission tomography. Circulation.

[7] Schwitter J., DeMarco T., Kneifel S., von Schulthess G.K., Jorg M.C., Arheden H., Ruhm S., Stumpe K., Buck A., Parmley W.W. (2000). Magnetic resonance-based assessment of global coronary flow and flow reserve and its relation to left ventricular functional parameters: A comparison with positron emission tomography. Circulation.

[8] Vogel R., Indermuhle A., Reinhardt J., Meier P., Siegrist P.T., Namdar M., Kaufmann P.A., Seiler C. (2005). The quantification of absolute myocardial perfusion in humans by contrast echocardiography: Algorithm and validation. J. Am. Coll. Cardiol..

[9] Di Carli M.F., Lipton M.J. (2007). Cardiac PET and PET/CT Imaging.

[10] Tillisch J., Brunken R., Marshall R., Schwaiger M., Mandelkern M., Phelps M., Schelbert H. (1986). Reversibility of cardiac wall-motion abnormalities predicted by positron tomography. N. Engl. J. Med..

[11] Schinkel A.F., Bax J.J., Poldermans D., Elhendy A., Ferrari R., Rahimtoola S.H. (2007). Hibernating myocardium: Diagnosis and patient outcomes. Curr. Probl. Cardiol..

[12] Beanlands R.S., Nichol G., Huszti E., Humen D., Racine N., Freeman M., Gulenchyn K.Y., Garrard L., deKemp R., Guo A. (2007). F-18-fluorodeoxyglucose positron emission tomography imaging-assisted management of patients with severe left ventricular dysfunction and suspected coronary disease: A randomized, controlled trial (PARR-2). J. Am. Coll. Cardiol..

[13] Camici P., Ferrannini E., Opie L.H. (1989). Myocardial metabolism in ischemic heart disease: Basic principles and application to imaging by positron emission tomography. Prog. Cardiovasc. Dis..

[14] Wijns W., Vatner S.F., Camici P.G. (1998). Hibernating myocardium. N. Engl. J. Med..

[15] Knuuti J., Schelbert H.R., Bax J.J. (2002). The need for standardisation of cardiac FDG PET imaging in the evaluation of myocardial viability in patients with chronic ischaemic left ventricular dysfunction. Eur. J. Nucl. Med. Mol. Imaging.

[16] Phelps M.E., Hoffman E.J., Selin C., Huang S.C., Robinson G., MacDonald N., Schelbert H., Kuhl D.E. (1978). Investigation of [^18^F]2-fluoro-2-deoxyglucose for the measure of myocardial glucose metabolism. J. Nucl. Med..

[17] Araujo L.I., Lammertsma A.A., Rhodes C.G., McFalls E.O., Iida H., Rechavia E., Galassi A., De Silva R., Jones T., Maseri A. (1991). Noninvasive quantification of regional myocardial blood flow in coronary artery disease with oxygen-15-labeled carbon dioxide inhalation and positron emission tomography. Circulation.

[18] Bergmann S.R., Herrero P., Markham J., Weinheimer C.J., Walsh M.N. (1989). Noninvasive quantitation of myocardial blood flow in human subjects with oxygen-15-labeled water and positron emission tomography. J. Am. Coll. Cardiol..

[19] Schelbert H.R., Phelps M.E., Hoffman E.J., Huang S.C., Selin C.E., Kuhl D.E. (1979). Regional myocardial perfusion assessed with N-13 labeled ammonia and positron emission computerized axial tomography. Am. J. Cardiol..

[20] Hutchins G.D., Schwaiger M., Rosenspire K.C., Krivokapich J., Schelbert H., Kuhl D.E. (1990). Noninvasive quantification of regional blood flow in the human heart using N-13 ammonia and dynamic positron emission tomographic imaging. J. Am. Coll. Cardiol..

[21] Herrero P., Markham J., Shelton M.E., Weinheimer C.J., Bergmann S.R. (1990). Noninvasive quantification of regional myocardial perfusion with rubidium-82 and positron emission tomography. Exploration of a mathematical model. Circulation.

[22] Nekolla S.G., Reder S., Saraste A., Higuchi T., Dzewas G., Preissel A., Huisman M., Poethko T., Schuster T., Yu M. (2009). Evaluation of the novel myocardial perfusion positron-emission tomography tracer 18F-BMS-747158-02: Comparison to ^13^N-ammonia and validation with microspheres in a pig model. Circulation.

[23] Maddahi J., Czernin J., Lazewatsky J., Huang S.C., Dahlbom M., Schelbert H., Sparks R., Ehlgen A., Crane P., Zhu Q. (2011). Phase I, first-in-human study of BMS747158, a novel 18F-labeled tracer for myocardial perfusion PET: Dosimetry, biodistribution, safety, and imaging characteristics after a single injection at rest. J. Nucl. Med..

[24] Berman D.S., Maddahi J., Tamarappoo B.K., Czernin J., Taillefer R., Udelson J.E., Gibson C.M., Devine M., Lazewatsky J., Bhat G. (2013). Phase II safety and clinical comparison with single-photon emission computed tomography myocardial perfusion imaging for detection of coronary artery disease: Flurpiridaz F 18 positron emission tomography. J. Am. Coll. Cardiol..

[25] Uren N.G., Melin J.A., De Bruyne B., Wijns W., Baudhuin T., Camici P.G. (1994). Relation between myocardial blood flow and the severity of coronary-artery stenosis. N. Engl. J. Med..

[26] Kaufmann P.A., Gnecchi-Ruscone T., Yap J.T., Rimoldi O., Camici P.G. (1999). Assessment of the reproducibility of baseline and hyperemic myocardial blood flow measurements with 15O-labeled water and PET. J. Nucl. Med..

[27] Kaufmann P.A., Gnecchi-Ruscone T., di Terlizzi M., Schafers K.P., Luscher T.F., Camici P.G. (2000). Coronary heart disease in smokers: Vitamin C restores coronary microcirculatory function. Circulation.

[28] Gaemperli O., Schepis T., Koepfli P., Siegrist P.T., Fleischman S., Nguyen P., Olmsted A., Wang W., Lieu H., Kaufmann P.A. (2008). Interaction of caffeine with regadenoson-induced hyperemic myocardial blood flow as measured by positron emission tomography: A randomized, double-blind, placebo-controlled crossover trial. J. Am. Coll. Cardiol..

[29] Wyss C.A., Koepfli P., Mikolajczyk K., Burger C., von Schulthess G.K., Kaufmann P.A. (2003). Bicycle exercise stress in PET for assessment of coronary flow reserve: Repeatability and comparison with adenosine stress. J. Nucl. Med..

[30] Maddahi J., Packard R.R. (2014). Cardiac PET perfusion tracers: Current status and future directions. Semin. Nucl. Med..

[31] Schafers K.P., Spinks T.J., Camici P.G., Bloomfield P.M., Rhodes C.G., Law M.P., Baker C.S., Rimoldi O. (2002). Absolute quantification of myocardial blood flow with H(2)(15)O and 3-dimensional PET: An experimental validation. J. Nucl. Med..

[32] Bol A., Melin J.A., Vanoverschelde J.L., Baudhuin T., Vogelaers D., De Pauw M., Michel C., Luxen A., Labar D., Cogneau M. (1993). Direct comparison of [13N]ammonia and [15O]water estimates of perfusion with quantification of regional myocardial blood flow by microspheres. Circulation.

[33] Bergmann S.R., Fox K.A., Rand A.L., McElvany K.D., Welch M.J., Markham J., Sobel B.E. (1984). Quantification of regional myocardial blood flow in vivo with H215O. Circulation.

[34] Selwyn A.P., Allan R.M., L’Abbate A., Horlock P., Camici P., Clark J., O’Brien H.A., Grant P.M. (1982). Relation between regional myocardial uptake of rubidium-82 and perfusion: Absolute reduction of cation uptake in ischemia. Am. J. Cardiol..

[35] Lautamaki R., George R.T., Kitagawa K., Higuchi T., Merrill J., Voicu C., DiPaula A., Nekolla S.G., Lima J.A., Lardo A.C. (2009). Rubidium-82 PET-CT for quantitative assessment of myocardial blood flow: Validation in a canine model of coronary artery stenosis. Eur. J. Nucl. Med. Mol. Imaging.

[36] Herrero P., Hartman J.J., Green M.A., Anderson C.J., Welch M.J., Markham J., Bergmann S.R. (1996). Regional myocardial perfusion assessed with generator-produced copper-62-PTSM and PET. J. Nucl. Med..

[37] Fukushima K., Javadi M.S., Higuchi T., Lautamaki R., Merrill J., Nekolla S.G., Bengel F.M. (2011). Prediction of short-term cardiovascular events using quantification of global myocardial flow reserve in patients referred for clinical 82Rb PET perfusion imaging. J. Nucl. Med..

[38] Ziadi M.C., Dekemp R.A., Williams K.A., Guo A., Chow B.J., Renaud J.M., Ruddy T.D., Sarveswaran N., Tee R.E., Beanlands R.S. (2011). Impaired myocardial flow reserve on rubidium-82 positron emission tomography imaging predicts adverse outcomes in patients assessed for myocardial ischemia. J. Am. Coll. Cardiol..

[39] Farhad H., Dunet V., Bachelard K., Allenbach G., Kaufmann P.A., Prior J.O. (2013). Added prognostic value of myocardial blood flow quantitation in rubidium-82 positron emission tomography imaging. Eur. Heart J. Cardiovasc. Imaging.

[40] Murthy V.L., Naya M., Foster C.R., Hainer J., Gaber M., Di Carli G., Blankstein R., Dorbala S., Sitek A., Pencina M.J. (2011). Improved cardiac risk assessment with noninvasive measures of coronary flow reserve. Circulation.

[41] Muzik O., Beanlands R.S., Hutchins G.D., Mangner T.J., Nguyen N., Schwaiger M. (1993). Validation of nitrogen-13-ammonia tracer kinetic model for quantification of myocardial blood flow using PET. J. Nucl. Med..

[42] Bellina C.R., Parodi O., Camici P., Salvadori P.A., Taddei L., Fusani L., Guzzardi R., Klassen G.A., L’Abbate A.L., Donato L. (1990). Simultaneous in vitro and in vivo validation of nitrogen-13-ammonia for the assessment of regional myocardial blood flow. J. Nucl. Med..

[43] Choi Y., Huang S.C., Hawkins R.A., Kim J.Y., Kim B.T., Hoh C.K., Chen K., Phelps M.E., Schelbert H.R. (1999). Quantification of myocardial blood flow using 13N-ammonia and PET: Comparison of tracer models. J. Nucl. Med..

[44] Chow B.J., Beanlands R.S., Lee A., DaSilva J.N., deKemp R.A., Alkahtani A., Ruddy T.D. (2006). Treadmill exercise produces larger perfusion defects than dipyridamole stress N-13 ammonia positron emission tomography. J. Am. Coll. Cardiol..

[45] Huisman M.C., Higuchi T., Reder S., Nekolla S.G., Poethko T., Wester H.J., Ziegler S.I., Casebier D.S., Robinson S.P., Schwaiger M. (2008). Initial characterization of an 18F-labeled myocardial perfusion tracer. J. Nucl. Med..

[46] Kim D.Y., Kim H.S., Le U.N., Jiang S.N., Kim H.J., Lee K.C., Woo S.K., Chung J., Kim H.S., Bom H.S. (2012). Evaluation of a mitochondrial voltage sensor, (18F-fluoropentyl)triphenylphosphonium cation, in a rat myocardial infarction model. J. Nucl. Med..

[47] Kim D.Y., Kim H.J., Yu K.H., Min J.J. (2012). Synthesis of [^18^F]-labeled (2-(2-fluoroethoxy)ethyl)tris(4-methoxyphenyl)phosphonium cation as a potential agent for positron emission tomography myocardial imaging. Nucl. Med. Biol..

[48] Kim D.Y., Kim H.J., Yu K.H., Min J.J. (2012). Synthesis of [^18^F]-labeled (6-fluorohexyl)triphenylphosphonium cation as a potential agent for myocardial imaging using positron emission tomography. Bioconj. Chem..

[49] Dilsizian V., Taillefer R. (2012). Journey in evolution of nuclear cardiology: Will there be another quantum leap with the F-18-labeled myocardial perfusion tracers?. JACC Cardiovasc. Imaging.

[50] Rischpler C., Park M.J., Fung G.S., Javadi M., Tsui B.M., Higuchi T. (2012). Advances in PET myocardial perfusion imaging: F-18 labeled tracers. Ann. Nucl. Med..

[51] Nekolla S.G., Saraste A. (2011). Novel F-18-labeled PET myocardial perfusion tracers: Bench to bedside. Curr. Cardiol. Rep..

[52] Camici P.G., Prasad S.K., Rimoldi O.E. (2008). Stunning, hibernation, and assessment of myocardial viability. Circulation.

[53] Barnes E., Dutka D.P., Khan M., Camici P.G., Hall R.J. (2002). Effect of repeated episodes of reversible myocardial ischemia on myocardial blood flow and function in humans. Am. J. Physiol. Heart Circ. Physiol..

[54] Pagano D., Fath-Ordoubadi F., Beatt K.J., Townend J.N., Bonser R.S., Camici P.G. (2001). Effects of coronary revascularisation on myocardial blood flow and coronary vasodilator reserve in hibernating myocardium. Heart.

[55] Underwood S.R., Bax J.J., vom Dahl J., Henein M.Y., Knuuti J., van Rossum A.C., Schwarz E.R., Vanoverschelde J.L., van der Wall E.E., Wijns W. (2004). Imaging techniques for the assessment of myocardial hibernation. Report of a Study Group of the European Society of Cardiology. Eur. Heart J..

[56] Desideri A., Cortigiani L., Christen A.I., Coscarelli S., Gregori D., Zanco P., Komorovsky R., Bax J.J. (2005). The extent of perfusion-F18-fluorodeoxyglucose positron emission tomography mismatch determines mortality in medically treated patients with chronic ischemic left ventricular dysfunction. J. Am. Coll. Cardiol..

[57] Cleland J.G., Calvert M., Freemantle N., Arrow Y., Ball S.G., Bonser R.S., Chattopadhyay S., Norell M.S., Pennell D.J., Senior R. (2011). The Heart Failure Revascularisation Trial (HEART). Eur. J. Heart Fail..

[58] Bonow R.O., Maurer G., Lee K.L., Holly T.A., Binkley P.F., Desvigne-Nickens P., Drozdz J., Farsky P.S., Feldman A.M., Doenst T. (2011). Myocardial viability and survival in ischemic left ventricular dysfunction. N. Engl. J. Med..

[59] Shah B.N., Khattar R.S., Senior R. (2013). The hibernating myocardium: Current concepts, diagnostic dilemmas, and clinical challenges in the post-STICH era. Eur. Heart J..

[60] Perrone-Filardi P., Pinto F.J. (2012). Looking for myocardial viability after a STICH trial: Not enough to close the door. J. Nucl. Med..

[61] Allman K.C., Shaw L.J., Hachamovitch R., Udelson J.E. (2002). Myocardial viability testing and impact of revascularization on prognosis in patients with coronary artery disease and left ventricular dysfunction: A meta-analysis. J. Am. Coll. Cardiol..

[62] Ponikowski P., Voors A.A., Anker S.D., Bueno H., Cleland J.G., Coats A.J., Falk V., Gonzalez-Juanatey J.R., Harjola V.P., Jankowska E.A. (2016). 2016 ESC Guidelines for the Diagnosis and Treatment of Acute and Chronic Heart Failure. Rev. Esp. Cardiol..

[63] Bax J.J., Visser F.C., Poldermans D., Elhendy A., Cornel J.H., Boersma E., Valkema R., Van Lingen A., Fioretti P.M., Visser C.A. (2001). Relationship between preoperative viability and postoperative improvement in LVEF and heart failure symptoms. J. Nucl. Med..

[64] Beanlands R.S., Ruddy T.D., deKemp R.A., Iwanochko R.M., Coates G., Freeman M., Nahmias C., Hendry P., Burns R.J., Lamy A. (2002). Positron emission tomography and recovery following revascularization (PARR-1): The importance of scar and the development of a prediction rule for the degree of recovery of left ventricular function. J. Am. Coll. Cardiol..

[65] Ling L.F., Marwick T.H., Flores D.R., Jaber W.A., Brunken R.C., Cerqueira M.D., Hachamovitch R. (2013). Identification of therapeutic benefit from revascularization in patients with left ventricular systolic dysfunction: Inducible ischemia versus hibernating myocardium. Circ. Cardiovasc. Imaging.

[66] Namdar M., Rager O., Priamo J., Frei A., Noble S., Amzalag G., Ratib O., Nkoulou R. (2018). Prognostic value of revascularising viable myocardium in elderly patients with stable coronary artery disease and left ventricular dysfunction: A PET/CT study. Int. J. Cardiovasc. Imaging.

[67] Machac J. (2005). Cardiac positron emission tomography imaging. Semin. Nucl. Med..

[68] Schindler T.H., Schelbert H.R., Quercioli A., Dilsizian V. (2010). Cardiac PET imaging for the detection and monitoring of coronary artery disease and microvascular health. JACC Cardiovasc. Imaging.

[69] Schindler T.H., Zhang X.L., Vincenti G., Mhiri L., Lerch R., Schelbert H.R. (2007). Role of PET in the evaluation and understanding of coronary physiology. J. Nucl. Cardiol..

[70] Camici P.G., Crea F. (2007). Coronary microvascular dysfunction. N. Engl. J. Med..

[71] Neglia D., Michelassi C., Trivieri M.G., Sambuceti G., Giorgetti A., Pratali L., Gallopin M., Salvadori P., Sorace O., Carpeggiani C. (2002). Prognostic role of myocardial blood flow impairment in idiopathic left ventricular dysfunction. Circulation.

[72] Shaw L.J., Min J.K., Hachamovitch R., Peterson E.D., Hendel R.C., Woodard P.K., Berman D.S., Douglas P.S. (2010). Cardiovascular imaging research at the crossroads. JACC Cardiovasc. Imaging.

[73] Hachamovitch R., Kang X., Amanullah A.M., Abidov A., Hayes S.W., Friedman J.D., Cohen I., Thomson L.E., Germano G., Berman D.S. (2009). Prognostic implications of myocardial perfusion single-photon emission computed tomography in the elderly. Circulation.

[74] Herzog B.A., Husmann L., Valenta I., Gaemperli O., Siegrist P.T., Tay F.M., Burkhard N., Wyss C.A., Kaufmann P.A. (2009). Long-term prognostic value of 13N-ammonia myocardial perfusion positron emission tomography added value of coronary flow reserve. J. Am. Coll. Cardiol..

[75] Schindler T.H., Campisi R., Dorsey D., Prior J.O., Olschewski M., Sayre J., Schelbert H.R. (2009). Effect of hormone replacement therapy on vasomotor function of the coronary microcirculation in post-menopausal women with medically treated cardiovascular risk factors. Eur. Heart J..

[76] Schindler T.H., Cardenas J., Prior J.O., Facta A.D., Kreissl M.C., Zhang X.L., Sayre J., Dahlbom M., Licinio J., Schelbert H.R. (2006). Relationship between increasing body weight, insulin resistance, inflammation, adipocytokine leptin, and coronary circulatory function. J. Am. Coll. Cardiol..

[77] Lima R.S., Watson D.D., Goode A.R., Siadaty M.S., Ragosta M., Beller G.A., Samady H. (2003). Incremental value of combined perfusion and function over perfusion alone by gated SPECT myocardial perfusion imaging for detection of severe three-vessel coronary artery disease. J. Am. Coll. Cardiol..

[78] Berman D.S., Kang X., Slomka P.J., Gerlach J., de Yang L., Hayes S.W., Friedman J.D., Thomson L.E., Germano G. (2007). Underestimation of extent of ischemia by gated SPECT myocardial perfusion imaging in patients with left main coronary artery disease. J. Nucl. Cardiol..

[79] Smith W.H., Kastner R.J., Calnon D.A., Segalla D., Beller G.A., Watson D.D. (1997). Quantitative gated single photon emission computed tomography imaging: A counts-based method for display and measurement of regional and global ventricular systolic function. J. Nucl. Cardiol..

[80] Gould K.L. (2009). Does coronary flow trump coronary anatomy?. JACC Cardiovasc. Imaging.

[81] Gould K.L. (2009). Coronary flow reserve and pharmacologic stress perfusion imaging: Beginnings and evolution. JACC Cardiovasc. Imaging.

[82] Dayanikli F., Grambow D., Muzik O., Mosca L., Rubenfire M., Schwaiger M. (1994). Early detection of abnormal coronary flow reserve in asymptomatic men at high risk for coronary artery disease using positron emission tomography. Circulation.

[83] Gimelli A., Schneider-Eicke J., Neglia D., Sambuceti G., Giorgetti A., Bigalli G., Parodi G., Pedrinelli R., Parodi O. (1998). Homogeneously reduced versus regionally impaired myocardial blood flow in hypertensive patients: Two different patterns of myocardial perfusion associated with degree of hypertrophy. J. Am. Coll. Cardiol..

[84] Di Carli M.F., Janisse J., Grunberger G., Ager J. (2003). Role of chronic hyperglycemia in the pathogenesis of coronary microvascular dysfunction in diabetes. J. Am. Coll. Cardiol..

[85] Tio R.A., Dabeshlim A., Siebelink H.M., de Sutter J., Hillege H.L., Zeebregts C.J., Dierckx R.A., van Veldhuisen D.J., Zijlstra F., Slart R.H. (2009). Comparison between the prognostic value of left ventricular function and myocardial perfusion reserve in patients with ischemic heart disease. J. Nucl. Med..

[86] Adenaw N., Salerno M. (2013). PET/MRI: Current state of the art and future potential for cardiovascular applications. J. Nucl. Cardiol..

[87] Valenta I., Quercioli A., Schindler T.H. (2014). Diagnostic value of PET-measured longitudinal flow gradient for the identification of coronary artery disease. JACC Cardiovasc. Imaging.

[88] Dey D., Diaz Zamudio M., Schuhbaeck A., Juarez Orozco L.E., Otaki Y., Gransar H., Li D., Germano G., Achenbach S., Berman D.S. (2015). Relationship between Quantitative Adverse Plaque Features from Coronary Computed Tomography Angiography and Downstream Impaired Myocardial Flow Reserve by 13N-Ammonia Positron Emission Tomography: A Pilot Study. Circul. Cardiovasc. Imaging.

[89] Pelizzari C.A., Chen G.T., Spelbring D.R., Weichselbaum R.R., Chen C.T. (1989). Accurate three-dimensional registration of CT, PET, and/or MR images of the brain. J. Comput. Assist. Tomogr..

[90] Woods R.P., Mazziotta J.C., Cherry S.R. (1993). MRI-PET registration with automated algorithm. J. Comput. Assist. Tomogr..

[91] Pichler B.J., Judenhofer M.S., Catana C., Walton J.H., Kneilling M., Nutt R.E., Siegel S.B., Claussen C.D., Cherry S.R. (2006). Performance test of an LSO-APD detector in a 7-T MRI scanner for simultaneous PET/MRI. J. Nucl. Med..

[92] Krizsan A.K., Lajtos I., Dahlbom M., Daver F., Emri M., Kis S.A., Opposits G., Pohubi L., Potari N., Hegyesi G. (2015). Promising Future: Comparable Imaging Capability of MRI-Compatible Silicon Photomultiplier and Conventional Photosensor Preclinical PET Systems. J. Nucl. Med..

[93] Robson P.M., Dey D., Newby D.E., Berman D., Li D., Fayad Z.A., Dweck M.R. (2017). MR/PET Imaging of the Cardiovascular System. JACC Cardiovasc. Imaging.

[94] Munoz C., Kunze K.P., Neji R., Vitadello T., Rischpler C., Botnar R.M., Nekolla S.G., Prieto C. (2018). Motion-corrected whole-heart PET-MR for the simultaneous visualisation of coronary artery integrity and myocardial viability: An initial clinical validation. Eur. J. Nucl. Med. Mol. Imaging.

